# Acute Exposure to Simulated Nocturnal Train Noise Leads to Impaired Sleep Quality and Endothelial Dysfunction in Young Healthy Men and Women: A Sex-Specific Analysis

**DOI:** 10.3390/ijerph192113844

**Published:** 2022-10-25

**Authors:** Omar Hahad, Johannes Herzog, Martin Röösli, Frank P. Schmidt, Andreas Daiber, Thomas Münzel

**Affiliations:** 1 Department of Cardiology, Cardiology I, University Medical Center of the Johannes Gutenberg-University Mainz, 55131 Mainz, Germany; 2 German Center for Cardiovascular Research (DZHK), Partner Site Rhine-Main, 55131 Mainz, Germany; 3 Leibniz Institute for Resilience Research (LIR), 55131 Mainz, Germany; 4 Swiss Tropical and Public Health Institute (Swiss TPH), 4123 Allschwil, Switzerland; 5 Faculty of Science, University of Basel, 4001 Basel, Switzerland

**Keywords:** environmental risk factor, train noise exposure, endothelial function, sleep quality, vitamin C

## Abstract

A series of human field studies demonstrated that simulated nocturnal traffic noise exposure impaired sleep quality and endothelial function, which could be significantly improved after intake of vitamin C in case of endothelial function. However, it remains unclear whether these changes follow a sex-specific pattern. Thus, we aimed to analyze the effect of simulated nocturnal train noise exposure on sleep quality, endothelial function and its associated changes after vitamin C intake, and other hemodynamic and biochemical parameters in young healthy men and women. We used data from a randomized crossover study, wherein 70 healthy volunteers (50% women) were each exposed to one control pattern (regular background noise) and two different train noise scenarios (30 or 60 train noise events per night, with average sound pressure levels of 52 and 54 dB(A), respectively, and peak sound level of 73–75 dB(A)) in their homes for three nights. After each night, participants visited the study center for the measurement of endothelial function as well as other hemodynamic and biochemical parameters. Sleep quality measured via self-report was significantly impaired after noise 30 and noise 60 nights in both men and women (*p* < 0.001 vs. control). Likewise, endothelial function measured by flow-mediated dilation (FMD) was significantly impaired after noise 30 and noise 60 nights in both men and women (*p* < 0.001 vs. control). While in women, vitamin C intake significantly improved FMD after both noise 30 and noise 60 study nights compared to control nights, no significant changes were observed in men. Exposure to simulated nocturnal train noise impairs sleep quality and endothelial function in both men and women, whereas a significant improvement of endothelial function after noise exposure and vitamin C intake could only be observed in women. These findings suggest for the first time that in men other mechanisms such as oxidative stress causing endothelial dysfunction may come into play.

## 1. Introduction

In the last years, strong evidence has emerged to demonstrate that traffic noise exposure is a risk factor for cardiovascular disease [1]. However, evidence from human studies that provide a mechanistic basis for the adverse cardiovascular effects of noise is scarce and comes mainly from a more recent series of field studies. In the first two field studies, we demonstrated that acute simulated nocturnal aircraft noise exposure induced vascular endothelial dysfunction, increased stress hormone release, worsened sleep quality, and/or increased blood pressure in healthy subjects and in patients with established coronary artery disease [2,3]. Importantly, endothelial dysfunction is considered as an early biomarker for subclinical atherosclerosis [4,5]. Therefore, its early detection may be useful in the risk stratification for cardiovascular disease. In a subsequent field study, we expanded our previous results by demonstrating that nocturnal train noise exposure leads to endothelial dysfunction and a reduction of sleep quality [6]. Interestingly, the administration of vitamin C caused a pronounced improvement of endothelial dysfunction after noise-exposed nights compared to the control night, pointing to an involvement of oxidative stress and inflammation in the pathogenesis of train-noise-induced endothelial dysfunction.

However, whether these traffic-noise-induced adverse cardiovascular consequences follow a sex-specific pattern is unclear. This is of special importance when it comes to targeted measures to reduce the burden of disease and disability by noise exposure. In general, the evaluation of sex-specific effects in the noise–disease relationship is largely lacking, and previous results are inconsistent. For example, an experimental study established that low-intensity noise exposure was associated with a more pronounced annoyance in women compared to men [7]. In contrast, sleep disturbance by traffic noise exposure may be more prominent in men than in women as established by Röösli et al. [8]. In support of this, a relationship between hypertension and residential aircraft noise exposure was observed only in men [9,10], whereas Babisch concluded there were no sex-specific differences in the relationship between traffic noise exposure and cardiovascular risk [11]. A recent study of myocardial infarction patients found that nocturnal traffic noise exposure (L_night_) was associated with an increased atherothrombotic risk in men but not in women [12]. Thus, the sex-specific analysis of underlying central pathophysiological mechanisms suggested for the noise–disease relationship may further help to distinguish between the noise effects in men and women.

With the present additional analysis, we sought to determine a potential sex-specific effect of simulated nocturnal train noise exposure on sleep quality, endothelial function and its associated changes after oral vitamin C intake, and other hemodynamic and biochemical parameters in young healthy men and women using data from the ZuG (Zuglärm und Gefäßfunktion—Train Noise and Vascular Function) study.

## 2. Methods

### 2.1. ZuG Study

The conception and the design of the ZuG study have been described in detail previously [6]. Briefly, the ZuG study is a randomized, double-blind, crossover study including 70 young, healthy, nonsmoking adults (50% women), which was conducted at the Department of Cardiology at the University Medical Center in Mainz, Germany. Recruitment took place between April 2017 and June 2018. Potential participants were found through social media and the distribution of flyers and posters in the region of Mainz, Germany. All participants underwent three study nights with three different noise scenarios. In the morning after each study night, they went to the study center for a comprehensive set of examinations. All participants were exposed to the different noise scenarios in the participants’ own bedrooms in a randomized manner. Study nights were prescheduled to ensure a minimum of three nonstudy nights between study nights and if possible, on the same weekday. In female participants, care was taken to synchronize study nights with the hormonal status.

The noise scenarios comprised a control (C), Noise 30, and Noise 60 study night. The control scenario contained no “playback-generated” noise events, but the participants were exposed to normal background noise present in their home environments (peak sound level of 65 dB(A)). The Noise 30 and Noise 60 study nights consisted of playback of train noise events with 30 and 60 noise events, respectively, each event with a peak sound level of 73–75 dB(A) and a duration of about 61–77 s. The noise events comprised the noise of passing trains. Four different train noise events were repeatedly played back with time between noise events following a long–short–long pattern. Noise events were recorded under controlled circumstances in the bedroom of a resident living near an important railway track of Germany located in the Mittelrheintal (Kamp-Bornhofen, near Boppard/Koblenz) being part of the Rhine-Alpine rail freight corridor Rotterdam–Genoa. Recordings took place between 10 p.m. and 6 a.m. with window tilted open and microphone placed 0.15 m above the headboard in an actual bedroom. Noise patterns were played back as MP3 files via customary portable audio systems, which were positioned 1 m above the floor at the end of the bed. To ensure compliance, sound pressure level (SPL) was continuously measured via class-2 sound level meters (Extech Datenlogger 407780A, 30–130 dB, Extech Datenlogger 407764, 3–130 dB), which were placed near the head of the participant.

Exclusion criteria consisted of the following: being an antitraffic noise activist, exposure to higher residential levels of nocturnal traffic noise as determined by noise maps (L_Aeq,22–6h_ > 45 dB(A) for rail traffic, road traffic, and aircraft noise), sleep disorder (indicated by a score >10 on the Pittsburgh Sleep Quality Index (PSQI) [13]) or psychiatric disorder assessed by the Mini-International Neuropsychiatric Interview (M.I.N.I.) [14], age-adjusted hearing loss of 30 dB(A) or more, obstructive sleep apnea in the screening test, current shift work and/or regular drug intake except oral contraceptives. Other hormonal therapies were excluded. Included participants were advised to refrain from intake of caffeine containing beverages, alcohol, and supplemental vitamins the day before, during, and in the morning after each study night. Participants were financially compensated when completing the study protocol. All procedures conducted in the ZuG study were in accordance with the declaration of Helsinki and were approved by the ethics committee of the Statutory Physician Board of the State Rhineland-Palatinate (permit number: 837.265.16 (10584)). Written informed consent was obtained prior to participation in the study.

### 2.2. Examinations

After each study night, participants were invited to the study center in a fasted state with all measurements conducted and samples collected before 10 a.m. During the study nights, a range of hemodynamic parameters including heart rate, blood pressure, and pulse transit time were continuously measured by wearing portable polygraphic screening devices (SOMNO Watch™plus or SOMNO touch™, SOMNOmedics GmbH, Randersacker, Germany) as described previously [2,3,6,15]. Devices were put on by the participants immediately before starting the study night after being carefully advised and trained at the study center. The flow-mediated dilatation (FMD) of the brachial artery was measured using standardized methods [16]. To evaluate the role of reactive oxygen species in the development of potential vascular side effects of noise exposure, 30 out of the 70 participants were consecutively chosen and orally administered 2 g of vitamin C directly after the initial measurement of FMD, which was followed 2 h later by a second FMD measurement (on the same day as the initial FMD measurement without vitamin C) using an exactly similar protocol for vitamin C administration as previously published [17]. A second FMD measurement was only performed in those participants receiving vitamin C. The relative change in FMD after vitamin C intake was calculated as the difference between the first and second FMD measurements. Afterwards, blood samples were drawn and immediately analyzed by our in-house clinical chemistry laboratory. For the measurement of global noise sensitivity, the Dortmund Noise Sensitivity Questionnaire (NoiSeQ) [18] was used. The sleep quality was evaluated by a visual analog scale (VAS) with the following question: “Overall, how well did you sleep last night?“ (VAS ranging from 0 cm meaning very good sleep quality to 10 cm meaning very bad sleep quality). A questionnaire comprising 19 items was used to assess the participants’ attitude toward train noise with higher values denoting a more negative attitude. Standard laboratory methods were used to determine stress hormone levels and biochemical parameters as described [6]. This included the measurement of C-reactive protein, neutrophils, cortisol, glucose, adrenaline, dopamine, and 8-isoprostane.

### 2.3. Statistical Analysis

Data were presented as mean ± standard deviation. Baseline/screening characteristics were presented sex-specifically with *p* values derived from Student’s *t* test or a Mann–Whitney test as appropriate for independent samples. To analyze the differences for the outcomes of interest, a repeated measures analysis of variance (ANOVA) or Friedman’s test, as appropriate, was used with the three noise scenarios as independent variables and with stratification by sex. We further tested the interaction term of sex and noise scenario in the ANOVA based on the whole sample for the outcomes of interest that delivered a main effect in the sex-specific analysis. In the next step, a *t* test for paired samples were used for pairwise comparison. All tests were two-sided and *p* values < 0.05 were considered as significant. The assumption of normality and variance homogeneity was checked. The statistical analysis was performed using IBM SPSS Statistics Version 27.

## 3. Results

### 3.1. Baseline/Screening Characteristics of the Study Sample

Table 1 displays the baseline/screening characteristics of the study sample. In total, 35 men (mean age 27 years) and 35 women (mean age 24 years) were enrolled. Four participants were excluded for not meeting the inclusion criteria (*n* = 1) or due to a withdrawal of written consent (*n* = 3). Women were significantly younger, had lower weight, body mass index, and creatinine values as well as higher HDL cholesterol compared to men. No significant differences were observed in the case of noise sensitivity, attitude towards train noise, and sleep quality.

### 3.2. Effects of Nocturnal Train Noise on Sleep Quality, Hemodynamic, and Laboratory Parameters in Women

**Figure 1:**
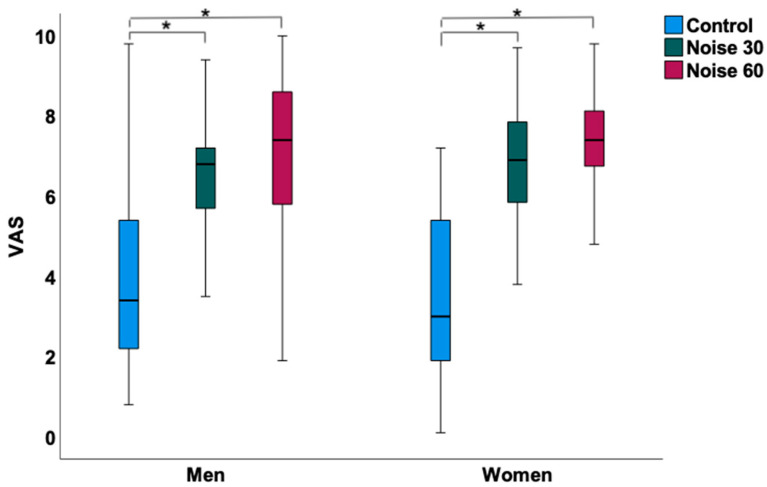
Effects of nocturnal train noise on sleep quality in men (*n* = 33) and women (*n* = 35) measured via visual analogue scale (VAS ranging from 0–10 cm with higher values indicating pronounced impaired sleep quality). Exposure to both train noise patterns impaired sleep quality, although no difference was observed between Noise 30 and Noise 60 study nights. * denotes *p* < 0.001. Box plots indicate minimum, maximum, 25th percentile, median, and 75th percentile.

**Figure 2:**
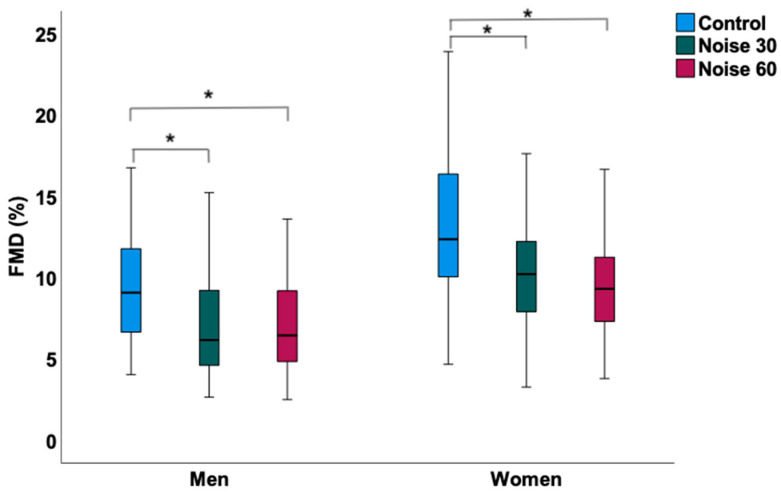
Flow-mediated dilation (FMD) upon nocturnal train noise exposure in men (*n* = 35) and women (*n* = 35). Exposure to both train noise patterns impaired endothelial function, although no difference was observed between Noise 30 and Noise 60 study nights. * denotes *p* < 0.001. Box plots indicate minimum, maximum, 25th percentile, median, and 75th percentile.

**Figure 3:**
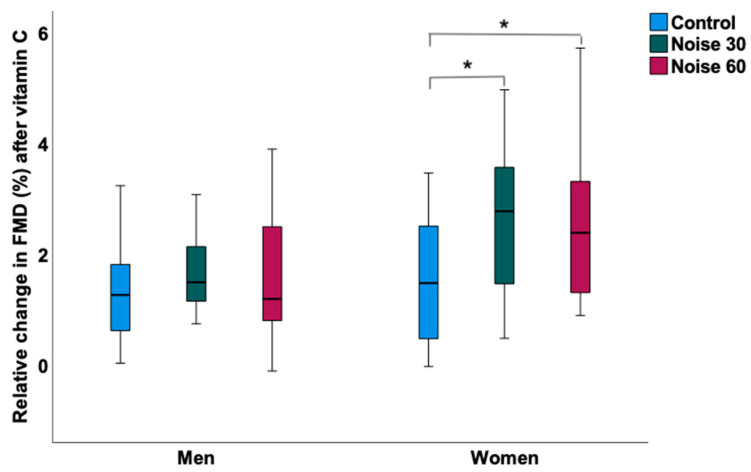
Determination of the effect of vitamin C on relative changes in endothelial function by nocturnal train noise exposure in men (*n* = 17) and women (*n* = 12). Flow-mediated dilation (FMD) was determined for Control, Noise 30, and Noise 60 study nights prior and post administration of vitamin C, which was used as an antioxidant drug to assess the impact of noise-triggered oxidative stress on endothelial function. Vitamin C significantly improved FMD in noise exposed study nights compared to control nights in women (*), although no difference was observed between Noise 30 and Noise 60 study nights. In men, vitamin C was not significantly associated with improved FMD in noise exposed study nights compared to control nights. Box plots indicate minimum, maximum, 25th percentile, median, and 75th percentile.

In women (Table 2), the L_Aeq_, the average sound pressure level, was 32.78 ± 3.91 dB(A) during the control nights, 52.58 ± 2.53 dB(A) during the Noise 30 nights and 54.18 ± 2.46 dB(A) during the Noise 60 nights. The peak levels of noise were lowest during the control nights with 66.44 ± 8.17 dB(A) and higher during the Noise 30 and 60 nights with 75.13 ± 3.09 dB(A) and 73.80 ± 2.78 dB(A), respectively. Self-reported sleep quality as determined by visual analog scale (ranging from 0–10 cm) was significantly disturbed after both Noise 30 (6.72 ± 1.9) and Noise 60 nights (7.26 ± 1.5) when compared to the control nights (3.43 ± 2.02), whereas no significant difference was observed between the noise nights (Figure 1). This was accompanied by significantly impaired endothelial function in both noise nights compared to the control nights displaying mean FMD values of 12.90 ± 4.76% after the control nights, 10.38 ± 3.81% after the Noise 30 nights and 9.85 ± 3.90% after the Noise 60 nights (Figure 2). However, no significant difference was observed between the noise nights. Concordantly, the administration of vitamin C improved FMD for all three exposure nights, while the relative change in FMD after the Noise 30 (2.59 ± 1.45%) and Noise 60 nights (2.50 ± 1.43%) was significantly higher than after the control nights (1.49 ± 1.13%) (Figure 3). No differences were observed in the case of heart rate, blood pressure, pulse transit time, and laboratory parameters.

### 3.3. Effects of Nocturnal Train Noise on Sleep Quality, Hemodynamic, and Laboratory Parameters in Men

In men (Table 3), the L_Aeq_, the average sound pressure level, was 33.81 ± 5.13 dB(A) during the control nights, 51.47 ± 2.76 dB(A) during the Noise 30 nights and 54.69 ± 2.81 dB(A) during the Noise 60 nights. The peak levels of noise were lowest during the control nights with 62.65 ± 8.56 dB(A) and higher during the Noise 30 and 60 nights with 74.58 ± 3.94 dB(A) and 74.79 ± 4.61 dB(A), respectively. Self-reported sleep quality was significantly disturbed after both Noise 30 (6.52 ± 1.71) and Noise 60 nights (7.12 ± 1.92) when compared to control nights (3.78 ± 2.12), whereas no significant difference was observed between the noise nights (Figure 1). This was accompanied by significantly impaired endothelial function in both noise nights compared to the control nights displaying mean FMD values of 9.56 ± 3.99% after the control nights, 7.04 ± 3.08% after the Noise 30 nights and 7.09 ± 3.01% after the Noise 60 nights (Figure 2). However, no significant difference was observed between the noise nights. In contrast to women, the relative change in FMD in response to vitamin C intake did not significantly differ after the Noise 30 (1.65 ± 0.69%) and Noise 60 nights (1.62 ± 1.15%) compared to the control nights (1.44 ± 1.08%) (Figure 3). No differences were observed in the case of heart rate, blood pressure, pulse transit time, and laboratory parameters except for adrenaline, which appeared to be significantly higher during the control nights compared to the Noise 30 nights. No significant interaction between the noise scenarios and sex was observed in the case of sleep quality, FMD, and relative change in FMD in response to vitamin C (data not shown).

## 4. Discussion

The results of the present study demonstrate, in line with our previous results, that simulated nocturnal train noise exposure is associated with endothelial dysfunction and a decreased self-reported sleep quality in both young healthy men and women, while Noise 30 and Noise 60 nights resulted in a comparable worsening of endothelial function and sleep quality. Importantly, the administration of vitamin C led to a pronounced improvement of endothelial function after the noise nights compared to the control nights but was restricted to women only.

The World Health Organization (WHO) recommends for average noise exposure (L_den_) to reduce noise levels produced by railway traffic below 54 dB, as railway noise above this level is associated with adverse health effects. For night noise exposure (L_night_), the conclusion is to reduce noise levels produced by railway traffic below 44 dB, as railway noise above this level is associated with adverse effects on sleep [19]. Importantly, according to European Environment Agency (EEA), more than 3 million people were exposed to railway noise levels above or equal to 55 dB L_den_, while nearly 2.5 million people were exposed to nocturnal railway noise levels exceeding or being equal to 50 dB L_night_ in 2017 in Germany [20]. In support of this, the results of our study clearly demonstrate that nocturnal train noise exposure that exceeds the recommended limit values by the WHO is associated with endothelial dysfunction accompanied by impaired sleep quality, which appeared to be stable in both men and women.

According to the noise reaction model put forward by Babisch, the so-called “indirect pathway” is the conceptual framework by which noise exposure negatively affects the cardiovascular system [21]. Herein, annoyance by chronic low-level noise exposure and its interference with daily routines and importantly sleep leads to an increased state of psycho-physiological arousal that is characterized by increased stress hormone levels, blood pressure, and heart rate. This, in turn, initiates and contributes to the development and acceleration of cardiovascular risk factors such as hypertension, arrhythmia, dyslipidemia, increased blood viscosity and blood glucose, and the activation of blood clotting factors, finally leading to manifest cardiovascular disease. However, the noise reaction model delivers no information concerning sex-specific noise health effects. The present study results only partly fit the rationale of the noise reaction model as we indeed observed interference with sleep and endothelial dysfunction in response to acute train noise exposure in both men and women, whereas we did not find any meaningful noise-induced changes in heart rate, blood pressure, stress hormones, and blood glucose. However, it is important to note that the “indirect pathway” of the noise reaction model rather refers to the long-term consequences of chronic exposure rather than acute noise effects, which was the subject of interest in the present study. The influence on these markers should be investigated in a field study with chronic train exposure.

There is high scientific plausibility to conclude that train noise exposure increases cardiovascular risk as suggested by recent large-scale epidemiological studies. In a nationwide cohort study from Denmark from Thacher et al., residential railway noise exposure at the least exposed façade was associated with a 28% higher risk of incident heart failure (hazard ratio (HR) 1.28, 95% CI 1.004–1.053) [22]. Within the same cohort, the authors further found suggestive evidence of an association between railway noise exposure and incident atrial fibrillation (incidence rate ratio (IRR) 1.017, 95 CI 1.007–1.026 for the most and IRR 1.035, 95% CI 1.021–1.050 for the least exposed façade), which was sensitive to further adjustment for air pollution (mean railway noise levels at the least exposed façade were ~46 dB L_den_ and at the most exposed façade were ~53 dB L_den_ [23]). Likewise, a nationwide cohort study from Switzerland demonstrated a higher risk of death from cardiovascular diseases (HR 1.013, 95% CI 1.10–1.017) and of death due to myocardial infarction (HR 1.020, 95% CI 1.010–1.030) in response to railway noise exposure. Moreover, blood-pressure-related, ischemic heart disease and all-stroke mortality were significantly associated with railway noise (mean railway noise levels ranged from 36.6 to 38.4 dB L_den_) [24]. In contrast, in a pooled study of nine Scandinavian cohorts from Roswall et al., no association was found between railway noise exposure and risk of incident stroke noise (mean railway noise levels ranged from 44.5 to 52.9 dB) [25]. Furthermore, Sørensen et al. did not find any evidence that railway noise exposure was associated with an increased risk of incident stroke in a nationwide study covering Denmark (with 339,915 individuals being exposed to >55 dB L_den_ railway noise levels) [26]. Interestingly, in a large Canadian cohort, the distance to major roads and highways were not associated with the incidence of myocardial infarction, whereas the proximity to railways was positively associated with incident MI (HR 1.07, 95% CI 1.01–1.14 for ≤100 vs. >1000 m, total noise levels ranged from ~44 to ~79 dB(A)) [27]. However, none of these studies investigated whether these associations followed a sex-specific pattern.

In our first field study from 2013, we established in a small group of healthy subjects (*n* = 5) that vitamin C treatment significantly improved endothelial function in subjects exposed to nocturnal aircraft noise [2]. In the ZuG study, we consecutively assigned participants to an acute vitamin C treatment and observed a significantly pronounced improvement in endothelial function in participants after the noise nights compared to the control nights, but which was restricted to women. In men, the relative change in FMD in response to vitamin C intake did not significantly differ after the Noise 30 and Noise 60 nights compared to the control nights, although from a descriptive point of view there was an improvement in all three scenarios. This observation has several potential implications. In line with previous results, the stronger improvement of endothelial function in women strongly suggests that the increased production of reactive oxygen species is an important underlying mechanism in noise-induced endothelial dysfunction supported by the stronger antioxidant effect of vitamin C in the presence of a higher burden of oxidative stress in response to nocturnal noise exposure. Interestingly, there are just a few sex-related observations in response to noise. For instance, Selander et al. observed in the HYENA trial, increases in morning saliva cortisol in women but not in men [28]. Moreover, a pooled analysis from seven European countries demonstrated a significant association between saliva cortisol levels and noise levels [29]. Importantly, Turner et al. observed a lower FMD in subjects with lupus erythematosus, which was associated with the duration of the corticosteroid therapy [30]. Although the plasma cortisol levels were not modified in the present studies, one might speculate that repeated assessments of saliva cortisol appears to be a more feasible and more preferable method to apply in field studies in noise research [31], which might have contributed to endothelial dysfunction and increased oxidative stress in women exposed to transportation noise.

It can be also concluded that in men different pathomechanisms are responsible for noise-induced endothelial dysfunction. In support of this, it is well-known that the hypothalamic–pituitary–adrenal (HPA) axis, which is stimulated by noise exposure leading to stress hormone release, follows a sex-specific response pattern and is influenced by the menstrual cycle and hormone status [32]. This would also implicate that the mechanisms of cardiovascular recovery in response to noise exposure may also differ between men and women. However, our present study cannot clarify this issue as our data only allow a hypothesis-generating approach.

In a recent systematic review by Rompel et al., the sex/gender differences in the health effects of environmental noise exposure on hypertension and ischemic heart disease were comprehensively analyzed [33]. In the majority of included studies, no effect modification by sex was found. The authors concluded that the reason for this could be either that biological sex is minorly important when it comes to noise-induced cardiovascular side effects or that gender-related differences (social, economic, and cultural factors in society) or the combination of both are also crucial in this setting. There are no studies providing gender-related analyses in the context of environmental noise exposure to date. Thus, dividing the study population in groups on the basis of a sex/gender variable, without an underlying concept, is not appropriate to identify sex differences or susceptible groups, as differences due to sex/gender variability within the groups might be greater than between them, as the authors conclude. Future studies should make efforts to disentangle between sex- and gender-related factors in the evaluation of health effects of noise.

The strengths of the present study include the novelty of analyzing sex-specific differences in the context of acute, controlled exposure to simulated nocturnal train noise, which has several advantages compared to epidemiological designs, where exposure misclassification is more likely. The limitations of the study include the nonrepresentativeness of the study sample, which comprised young and healthy men and women. We included young subjects without pre-existing chronic diseases and chronic exposure to higher noise levels. Thus, inclusion criteria were determined to result in a healthy study population and to avoid pre-existing confounding effects on FMD such as given by chronic noise exposure, smoking, sleep disorders, and other lifestyle factors known to have an impact on sleep quality, e.g., shift work. As a result, the findings of our work are not, or only in a limited fashion, transferable to nonhealthy or chronically noise-exposed subjects. Although we have previously shown that nocturnal aircraft noise exposure also leads to a significant impairment of FMD in patients with established coronary artery disease [3], further studies are needed to clarify this point. The experimental setup as a field study aimed to create ecologically valid conditions and thus to avoid a pure laboratory environment. However, this leads also to less experimental control over ambient conditions, sound levels, and external stimuli. The use of a VAS to determine self-reported sleep quality is limited as there are no validated cut-off values (or similar) available, thus making a clear interpretation rather difficult. The determination of objective measures of sleep quality would be beneficial at this point. A further major limitation includes the circumstance that we could not account for oral contraceptive use in women, which likely interfered with the present results. Furthermore, we did not include a control group of subjects receiving no vitamin C followed by a second FMD measurement, which would have allowed a direct comparison of the effect of receiving vitamin C vs. receiving no vitamin C on FMD after noise exposure. Both Noise 30 and Noise 60 nights were associated with a significant impairment of FMD compared to the control nights in both men and women. However, no difference was observed between the Noise 30 and Noise 60 nights. An explanation for this may include the resulting high sound pressure levels with peaks of 73–75 dB(A) and means of 51–54 dB(A), which are, for example, mostly higher than in our recent aircraft noise exposure study (peaks of 60 dB(A) and means of 43–46 dB(A)) in which we could observe a dose-dependent impairment of FMD [2]. Further experimental train noise studies should include a variation in exposure levels. Furthermore, the rather low sample size did not allow comprehensive, sophisticated analyses, specifically when it came to a subgroup analysis. Stress hormone levels are known to show large variations over the day and thus, the estimation of this parameter hours after awakening may not have been optimal. A higher variability in outcomes of interest may have occurred due to the field study design, in which participants were potentially exposed to a number of different pollutants on their way to the study center, e.g., noise and air pollution, that may have diluted the observed effects. The measurement of endothelial function in different vascular beds (micro- and macrovascular) would probably more precisely allow a more accurate assessment of total vascular damage.

## 5. Conclusions

In conclusion, our results demonstrated that nocturnal train noise exposure was associated with endothelial dysfunction and a decreased sleep quality in both men and women, whereas a relative improvement of endothelial function after vitamin C intake was only observed in women. Thus, our results might suggest for the first time that in men compared to women, other mechanisms such as oxidative stress causing endothelial dysfunction may come into play.

## Figures and Tables

**Table 1:** Baseline/screening characteristics of the study sample stratified by sex (N = 70).

	Women (*n* = 35)	Men (*n* = 35)	*p* Value
Age (years)	24.31 ± 4.44	27.17 ± 6.19	**0.030**
Weight (kg)	60.34 ± 6.82	76.66 ± 10.42	**<0.001**
BMI (kg/m^2^)	21.23 ± 2.18	23.42 ± 2.54	**<0.001**
NoiSeQ total (0–3)	1.18 ± 0.36	1.35 ± 0.38	0.072
NoiSeQ Sleep dimension (0–3)	0.99 ± 0.51	1.23 ± 0.65	0.101
PSQI (0–21)	5.23 ± 1.63	5.34 ± 2.15	0.803
Attitude towards train noise questionnaire (0–64)	31.60 ± 5.51	31.03 ± 7.05	0.709
CRP (mg/L)	1.65 ± 2.56	0.98 ± 1.77	0.148
HbA1c (%)	5.05 ± 0.21	5.05 ± 0.38	0.908
LDL (mg/dL)	92.63 ± 27.0	100.69 ± 24.74	0.197
HDL (mg/dL)	67.86 ± 13.70	55.83 ± 7.62	**<0.001**
Total cholesterol (mg/dL)	177.46 ± 32.08	177.37 ± 28.79	0.991
Triglycerides (mg/L)	84.89 ± 38.95	104.40 ± 45.89	0.059
Creatinine (mg/dL)	0.79 ± 0.16	0.93 ± 0.12	**<0.001**

Data are presented as mean ± standard deviation. Statistically significant *p* values (*p* < 0.05) are given in bold. BMI, body mass index; NoiSeQ, Dortmund Noise Sensitivity Questionnaire with a score of three representing greatest noise sensitivity; PSQI, Pittsburgh Sleep Quality Index with a higher score indicating worse sleep quality; attitude towards train noise questionnaire with higher values denoting a negative attitude; CRP, C-reactive protein; HbA1c, glycated hemoglobin; LDL, low density lipoprotein; HDL, high density lipoprotein.

**Table 2:** Effects of nocturnal train noise on sleep quality, hemodynamic, and laboratory parameters in women (*n* = 35).

	Control	Noise 30	Noise 60	*p* Value
Peak (dB(A))	66.44 ± 8.17	75.13 ± 3.09	73.80 ± 2.78	**<0.001**
L_Aeq_ (dB(A))	32.78 ± 3.91	52.58 ± 2.53	54.18 ± 2.36	**<0.001**
Sleep quality (VAS 0–10 cm)	3.43 ± 2.02	6.72 ± 1.9	7.26 ± 1.5	**<0.001**
*Hemodynamic parameters*				
FMD (%)	12.90 ± 4.76	10.38 ± 3.81	9.85 ± 3.90	**<0.001**
FMD relative change after vitamin C (%)	1.49 ± 1.13	2.59 ± 1.45	2.50 ± 1.43	**0.032**
HR mean (bpm)	62.2 ± 8.9	61.6 ± 8.3	62.2 ± 9.2	0.713
HR maximum (bpm)	108.1 ± 16.9	107.6 ± 15.6	109.9 ± 12.9	0.667
HR acceleration index	156.6 ± 114.5	229.0 ± 187.9	189.7 ± 151.8	0.092
BP systolic mean (mmHg)	108.8 ± 10.8	110.8 ± 10.0	106.6 ± 12.9	0.103
BP diastolic mean (mmHg)	69.55 ± 10.8	70.31 ± 11.1	69.7 ± 10.5	0.913
BP rise index	28.8 ± 37.7	33.7 ± 32.8	28.9 ± 31.0	0.915
PTT mean (m/s)	325.2 ± 19.7	325.7 ± 21.5	326.5 ± 21.6	0.744
PTT maximum (m/s)	362.8 ± 58.2	375.9 ± 23.2	374.3 ± 22.2	0.263
PTT minimum (m/s)	270.4 ± 24.1	267.4 ± 25.8	264.8 ± 22.2	0.634
*Laboratory parameters*				
C-reactive protein (mg/L)	3.20 ± 10.81	3.09 ± 11.1	1.30 ± 2.10	0.914
Neutrophils (%)	51.8 ± 9.6	52.3 ± 11.4	52.6 ± 9.8	0.867
Cortisol (μg/L)	17.92 ± 5.84	17.75 ± 6.5	17.03 ± 5.18	0.330
Glucose (mg/dL)	84.2 ± 4.9	83.1 ± 5.5	84.7 ± 4.9	0.104
Adrenaline (pg/mL)	28.0 ± 22.01	23.3 ± 17.5	24.0 ± 16.7	0.855
Dopamine (pg/mL)	11.63 ± 12.3	11.47 ± 9.4	11.47 ± 9.9	0.567
8-isoprostane (pg/mL)	39.4 ± 18.86	42.10 ± 23.9	39.2 ± 18.6	0.520

Data are presented as mean ± standard deviation. Statistically significant *p* values (*p* < 0.05) are given in bold. Peak (dB(A)), highest sound pressure level measured during study night; L_Aeq_ (dB(A)), long-term average equivalent continuous sound level during study night; FMD, flow-mediated dilation; HR, heart rate; VAS, visual analogue scale (high values denoting worsening of sleep quality); PTT, pulse transit time; BP, blood pressure.

**Table 3:** Effects of nocturnal train noise on sleep quality, hemodynamic, and laboratory parameters in men (*n* = 35).

	Control	Noise 30	Noise 60	*p* Value
Peak (dB(A))	62.65 ± 8.56	74.58 ± 3.94	74.79 ± 4.61	**<0.001**
L_Aeq_ (dB(A))	33.81 ± 5.13	51.47 ± 2.76	54.69 ± 2.81	**<0.001**
Sleep quality (VAS 0–10 cm)	3.78 ± 2.12	6.52 ± 1.71	7.12 ± 1.92	**<0.001**
*Hemodynamic parameters*				
FMD (%)	9.56 ± 3.99	7.04 ± 3.08	7.09 ± 3.01	**<0.001**
FMD relative change after vitamin C (%)	1.44 ± 1.08	1.65 ± 0.69	1.62 ± 1.15	0.768
HR mean (bpm)	56.9 ± 6.2	55.9 ± 7.1	56.5 ± 6.6	0.474
HR maximum (bpm)	101.2 ± 10.1	105.4 ± 18.0	104.7 ± 11.9	0.212
HR acceleration index	153.7 ± 169.9	128.0 ± 150.2	147.6 ± 140.1	0.114
BP systolic mean (mmHg)	121.5 ± 13.7	122.7 ± 13.9	121.3 ± 10.7	0.853
BP diastolic mean (mmHg)	76.13 ± 10.4	77.77 ± 8.2	75.57 ± 9.0	0.509
BP rise index	32.8 ± 41.6	28.8 ± 30.6	45.9 ± 52.9	0.717
PTT mean (m/s)	341.9 ± 14.8	339.0 ± 26.3	338.7 ± 15.9	0.516
PTT maximum (m/s)	383.4 ± 15.4	383.91 ± 13.5	376.3 ± 60.4	0.480
PTT minimum (m/s)	292.5 ± 23.7	280.6 ± 27.4	280.7 ± 28.2	0.052
*Laboratory parameters*				
C-reactive protein (mg/L)	0.77 ± 1.03	0.84 ± 1.18	0.96 ± 1.67	0.720
Neutrophils (%)	52.1 ± 7.9	52.7 ± 5.8	53.0 ± 6.8	0.716
Cortisol (μg/L)	13.0 ± 2.53	13.35 ± 2.6	13.27 ± 2.36	0.749
Glucose (mg/dL)	89.4 ± 6.3	90.0 ± 5.8	91.3 ± 5.9	0.158
Adrenaline (pg/mL)	26.03 ± 21.65	22.7 ± 24.3	25.1 ± 21.5	**0.024**
Dopamine (pg/mL)	8.0 ± 8.3	9.26 ± 8.9	8.17 ± 9.0	0.421
8-isoprostane (pg/mL)	38.8 ± 21.41	38.90 ± 20.8	41.1 ± 22.3	0.358

Data are presented as mean ± standard deviation. Statistically significant *p* values (*p* < 0.05) are given in bold. Peak (dB(A)), highest sound pressure level measured during study night; L_Aeq_ (dB(A)), long-term average equivalent continuous sound level during study night; FMD, flow-mediated dilation; HR, heart rate; VAS, visual analogue scale (high values denoting worsening of sleep quality); PTT, pulse transit time; BP, blood pressure.

## Data Availability

The datasets analyzed during the current study are available from the corresponding author upon reasonable request.
